# Septic pulmonary embolism associated with periodontal disease: a case report and literature review

**DOI:** 10.1186/s12879-019-3710-3

**Published:** 2019-01-21

**Authors:** Tsuyoshi Watanabe, Masamichi Yokoe, Yoshinori Noguchi

**Affiliations:** 1 Department of Rheumatology, Chubu Rosai Hospital, 2-10-15, Komei-cho, Minato-ku, Nagoya, Aichi 468-0008 Japan; 2 Division of General Internal Medicine, Nagoya Red Cross Daini Hospital, Nagoya, Aichi 466-8650 Japan; 3 Department of Rheumatology, Chubu Rosai Hospital, 1-10-6, Komei-cho, Minato-ku, Nagoya, 455-8530 Japan

**Keywords:** Periodontal disease, Septic pulmonary embolism, *Parvimonas micra*

## Abstract

**Background:**

Periodontal disease, including periodontitis, has been reported to be a rare cause of septic pulmonary embolism (SPE). It is however extremely difficult to isolate the causative pathogen of periodontal disease-associated SPE from blood cultures of these patients.

**Case presentation:**

In this study, an 85-year-old Japanese man was admitted with fever and worsening malaise. He was later noted to have multiple bilateral subpleural pulmonary nodules on chest computed tomography scan. After admission, *Parvimonas micra* (*P. micra*) was isolated from his blood culture. This was followed by a meticulous search for the primary source of SPE, focusing on the head and neck areas. Consequently, apical periodontitis and infratemporal fossa abscess were identified as the primary sources of SPE. Although *P. micra* is one of the most frequently detected bacteria in the infected root canals of teeth with chronic apical periodontitis, it has rarely been proven as a causative pathogen of periodontal disease-associated SPE.

**Conclusions:**

This case study demonstrated that periodontal disease is an important primary source of SPE and *P. micra* could be a causative pathogen of SPE.

## Background

Septic pulmonary embolism (SPE) is a rare disorder that generally presents with an insidious onset of fever, respiratory symptoms, and lung infiltrates. SPE is usually associated with tricuspid valve infectious endocarditis (IE), infected central venous catheters, septic thrombophlebitis including Lemierre’s syndrome, and skin and soft tissue infections [1]. Periodontal disease, including periodontitis, has been reported to be a less common but important cause of SPE [2, 3]. However, it is difficult to identify the causative pathogen of periodontal disease-associated SPE, likely due to inappropriate culturing and sample collection techniques [1, 2]. Oropharyngeal anaerobic organisms, except for *Fusobacterium* species, an important causative bacterium for Lemierre’s syndrome, grow infrequently in the blood cultures of SPE patients.

Here, we report a case of periodontal disease-associated SPE caused by *Parvimonas micra* (*P. micra*) which was identified by blood culture tests, and later successfully treated with a combination therapy of antibiotics along with aspiration and drainage of the concurrent infratemporal fossa abscess.

## Case presentation

**Fig. 1:**
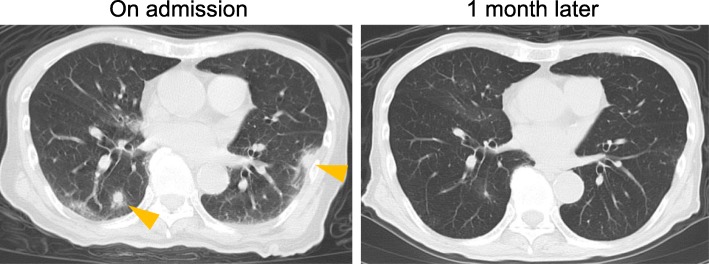
CT scans of septic pulmonary emboli. Chest CT images on admission (left panel). The arrowheads indicate septic embolic lesions. Subpleural ground glass opacity lesions were also present. Pulmonary lesions had resolved in a follow-up CT image at 1 month after the admission (right panel)

An 85-year-old Japanese man was admitted to our hospital with a two-day history of fever and worsening malaise. He denied respiratory tract symptoms. The patient had a medical history of diabetes mellitus (hemoglobin A1c level, 7.0%), but no past medical history of pulmonary disease. On admission, his vital signs were recorded as follows: heart rate, 90 beats/min; blood pressure, 123/77 mmHg; room-air oxygen saturation, 94%; respiratory rate, 19 breaths/min; and body temperature, 38.2 °C. Based on the laboratory test results, the patient was diagnosed with leukocytosis (12,000 cells/μL) and showed elevated levels of C-reactive protein (18.8 mg/dL); however, his liver and renal functions were normal. A chest radiograph showed multiple small pulmonary infiltrates in both lungs. Additional chest computed tomography (CT) scan revealed multiple bilateral pulmonary nodules mainly in subpleural areas (Fig. 1, left panel), suggesting the diagnosis of SPE.

**Fig. 2:**
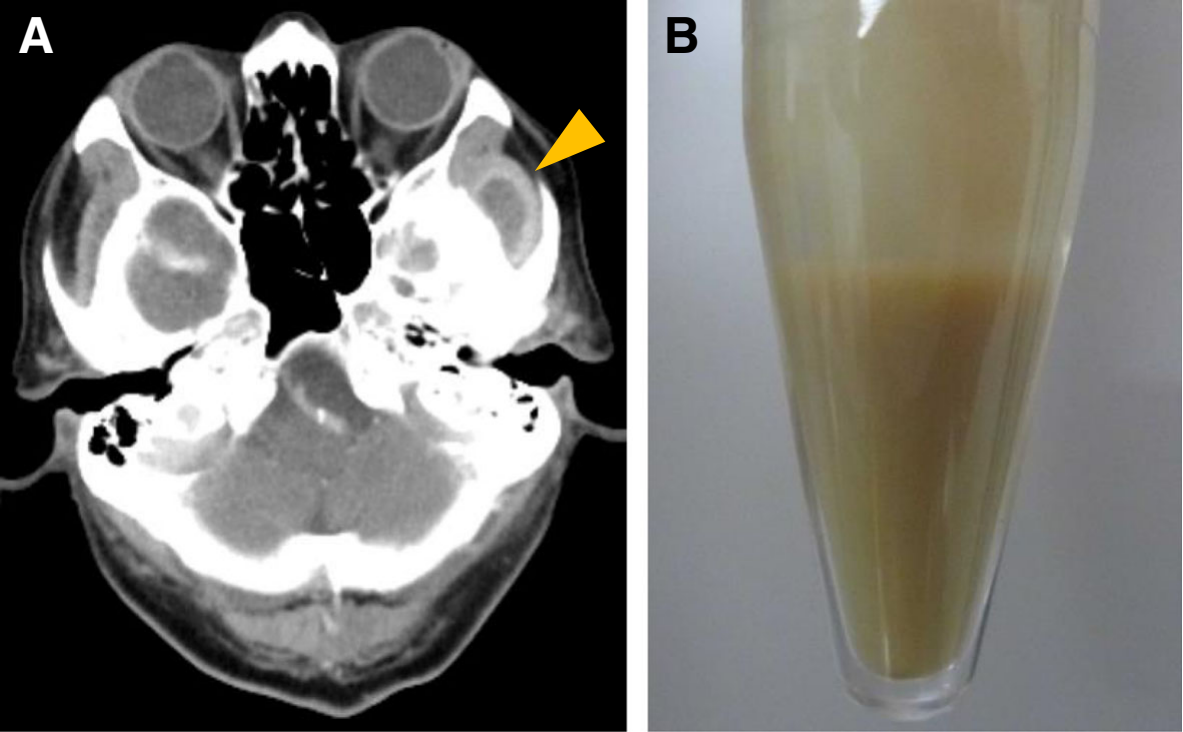
Images of infratemporal fossa abscess. **a** Contrast-enhanced head CT scan. Arrowheads indicates infratemporal fossa abscess. **b** Photograph of aspirated abscess

While searching for the primary source of infection, transthoracic echocardiography was performed multiple times (on the day of admission and 5 days after admission), and showed the absence of vegetation on the heart valves. Additionally, contrast-enhanced CT scan of the neck, chest, abdomen, and pelvis revealed neither an abscess nor suppurative thrombosis on the day of admission. After collection of the blood sample, empiric treatment with meropenem (1 g every 8 h) and vancomycin (1 g every 12 h) was initiated. On the 3rd day of hospital stay, the blood culture with a BacT/ALERT 3D system (BioMérieux, France) was found to be positive for *P. micra,* identified by RapID-ANA II system (Innovative Diagnostic Systems, Inc., Atlanta, Ga), a qualitative micromethod employing conventional and chromogenic substrates for the identification of anaerobic bacteria. The isolate was highly susceptible to all β-lactams, clindamycin, and carbapenems. Repeated physical assessment to detect the source of infection revealed mild tenderness on the left temple. Oral examination by a dentist showed periapical periodontitis at the root of the second left maxillary premolar. Further, contrast-enhanced facial CT scan identified an abscess in the infratemporal fossa (Fig. 2a), and thus puncture drainage of pus was performed on the 9th day of hospital stay (Fig. 2b). Following a review of susceptibility test results, we changed the antibiotics to ampicillin-sulbactam (3 g every 8 h), targeting *P. micra* and all possible anaerobic bacteria. Although Gram stain of the sample taken from the infratemporal fossa abscess showed positive staining for polymicrobial patterns of Gram-positive cocci and Gram-negative rod strains, only *Prevotella oris* (not *P. micra*) was isolated from culture of the abscess. The isolation same method was used for *Prevotella oris* and *P. micra*. Isolated *Prevotella oris* was susceptible to ampicillin-sulbactam, clindamycin, and carbapenems, but intermediately susceptible to penicillin and ampicillin. *P. micra* was not recovered from the infratemporal fossa abscess, probably due to the effect of ampicillin-sulbactam and difficulty in culturing anaerobic organisms.

**Fig. 3:**
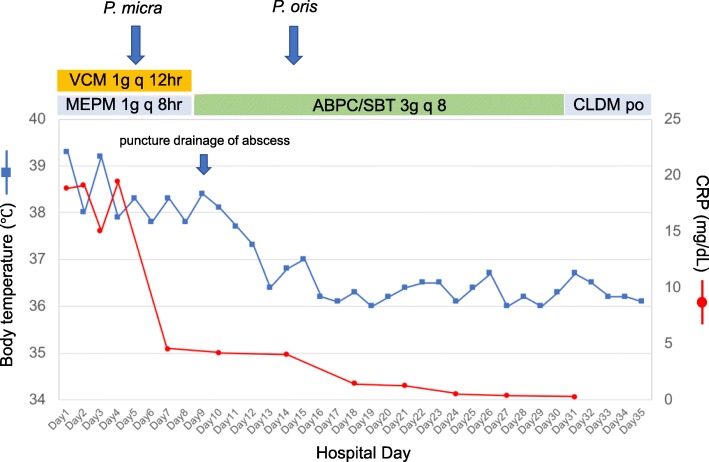
Clinical course until day 35. VCM vancomycin, MEPM meropenem, ABPC ampicillin, SBT sulbactam, CLDM clindamycin

Thus, we diagnosed this case as periodontal disease-associated SPE and infratemporal fossa abscess. Following combined treatment with ampicillin-sulbactam, tooth extraction, and puncture drainage of the infratemporal fossa abscess, the patient’s symptoms including fever and malaise gradually improved (Fig. 3). Intravenous antibiotic therapy with ampicillin-sulbactam was administered for 4 weeks, followed by continued oral treatment with clindamycin for 4 weeks. We confirmed complete remission of the lung lesions by CT scan (Fig. 1, right panel), and there has been no recurrence of symptoms after discontinuation of antibiotics.

## Discussion and conclusions

Herein, we present a case in which periodontal disease was the primary source of SPE and *P. micra* was identified as a causative pathogen of periodontal disease-associated SPE by blood culture.

Common causes of SPE include intravenous drug use, IE of the tricuspid valve, septic thrombophlebitis, indwelling intravascular catheters, and skin and soft tissue infection [1]. Periodontal disease, including periodontitis and peri-apical abscess has been reported to be infrequent but closely associated with SPE. According to a previous systematic review of SPE due to all causes [1], blood cultures dominantly grew methicillin-sensitive *Staphylococcus aureus* (MSSA) in 48 of 168 cases (28.6%) and methicillin-resistant *Staphylococcus aureus* (MRSA) in 27 of 168 cases (16.1%), and SPE was associated with a poor prognosis and 10.1% mortality. On the other hand, isolation of the causative pathogen has been rare for periodontal disease-associated SPE [2, 4]; hence, little is known about its etiology.

We searched PubMed and Ichushi-Web (Japan Medical Abstracts Society: JAMAS) databases for journal articles written in English and Japanese, using the search terms ‘Septic pulmonary embolism’ and ‘periodontal disease’ or ‘periodontitis’. Articles published from January 1, 1980 to September 1, 2018 were included. A literature review disclosed thirty-seven previously reported cases of periodontal disease-associated SPE including the present case (Table 1) [2, 3, 5–18]. The case definition for periodontal disease-associated SPE included: (A) focal or multifocal lung filtrates compatible with septic embolism to the lung, (B) presence of periodontal disease, and (C) absence of other active extrapulmonary infection as a potential cause of SPE, which were confirmed for each case of previously reported periodontal disease-associated SPE. The definition was a slightly modified version of those reported by Cook et al. [4] and Hatani et al. [5].

**Table 1:** Clinical summary of cases with periodontal disease-associated septic pulmonary embolism

Age	Sex	Local location	Culture^a^	Blood culture	Underlying disease	Immunocompromised	Prognosis	Reference
85	man	Periodontitis, temporal fossa abscess	*Prevotella oris*	*Provimonas micra*	Diabetes mellitus		alive	Present Case
56	man	Mandible and maxilla	*Streptococcus intermedius*	Negative	None		alive	[6]
50	man	Periodontal abscess	ND ^b^	Negative	Osler-Weber-Rendu disease		alive	[7]
53	man	Periodontitis	ND	ND	None		alive	[8]
67	man	Periodontitis	ND	Negative	Bronchial asthma	Prednisolone	alive	[8]
52	man	Periodontitis	ND	ND	Hypertensive nephropathy, renal transplant	Prednisolone, azathioprine	alive	[2]
70	woman	Periodontitis, pterygomandibular space abscess	ND	ND	None		alive	[9]
49	man	Periodontitis	ND	ND	Diabetes mellitus		alive	[10]
39	man	Periodontitis, periapical periodontitis	Negative	Negative	None		alive	[11]
24	woman	Periodontal abscess	ND	Gram positive cocci	Rheumatic mitral valve stenosis,congestive heart failure		alive	[12]
85	man	Periodontitis	ND	Negative	Hypertension		alive	[13]
61	woman	Periodontitis, masticator space abscess	*Streptococcus* species	ND	Diabetes mellitus		alive	[14]
81	woman	Impacted tooth	ND	ND	Hypertension		alive	[15]
53	man	Chronic periodontitis	ND	Negative	Hypertension		alive	[5]
75	woman	Periodontitis	ND	Negative	None		alive	[5]
52	man	Chronic periodontitis	ND	Negative	None		alive	[5]
54	man	Periodontal abscess	ND	Negative	Hypertension		alive	[5]
64	man	Periodontitis, Periodontal abscess	ND	Negative	Hypertension, mitral regurgitation		alive	[5]
72	woman	Periodontitis	ND	Negative	Hypertension		alive	[5]
65	man	Periodontal abscess	ND	Negative	None		alive	[5]
33	man	Periodontitis	ND	Negative	None		alive	[5]
57	man	Periodontitis	ND	Negative	Hypertension		alive	[5]
71	man	Periodontal abscess	ND	Negative	Hypertension, diabetes mellitus		alive	[5]
62	man	Periodontitis	ND	Negative	Hypertension		alive	[5]
68	man	Periodontitis	ND	Negative	Diabetes mellitus		alive	[5]
52	man	Periodontitis	ND	ND	Hypertension		alive	[16]
74	man	Periodontitis	ND	ND	None		alive	[16]
55	man	Periodontitis	ND	Negative	None		alive	[16]
59	man	Periodontitis	ND	Negative	Hypertension, Emphysema		alive	[16]
70	man	Periodontitis, periapical periodontitis	ND	Negative	Aortic aneurysm, Bladder stone		alive	[16]
65	man	Periodontitis	ND	Negative	Cerebral infarction		alive	[16]
59	man	Periodontitis	ND	Negative	Hyperlipidemia		alive	[16]
47	man	Periodontitis	ND	Negative	None		alive	[16]
56	man	Periodontitis	ND	Negative	None		alive	[16]
59	man	Perimandibular abscess	Negative	Negative	Diabetes mellitus		alive	[17]
64	man	Periodontitis	*Actinomyces* species	Negative	Diabetes mellitus		alive	[3]
77	man	Periodontitis	ND	*Peptostreptococcus* species,*Fusobacterium nucleatum*	Diabetes mellitus		alive	[18]

^a^culture finding of periodontal abscess

^b^ND, no data

The mean age of the patients with periodontal disease-associated SPE was 60.4 (range, 24–85) years. All cases were community-acquired. In addition, we confirmed a male preponderance of periodontal disease-associated SPE (83.7%). In previous studies, it was shown that men had higher prevalence and severity of periodontal destruction compared to women [19, 20]. The age group most commonly affected by periodontal disease-associated SPE was older than that of the entire cases of SPE [1, 4]. It was reported that the prevalence of periodontitis increases with age up to ages 55–59 years, with reduction in the older age population due to the loss of affected teeth [21]. Thus, it is possible that the middle-aged population and men are at risk of periodontal disease-associated SPE.

Culture findings of pus and blood were positive in only four (10.8%) and three (8.1%) cases of periodontal disease-associated SPE, respectively. Indigenous oral bacteria such as *P. micra*, *Prevotella oris*, *Streptococcus intermedius*, *Actinomyces* species, and *Peptostreptococcus* species were mainly isolated as causative pathogens. The isolation rate of the causative pathogen in periodontal disease-associated SPE was significantly lower than that of SPE due to common causes including intravenous drug use, IE of the tricuspid valve, and septic thrombophlebitis [1]. We speculate that this discrepancy may be due to difficulties in culturing fastidious bacteria from the oral cavity, inappropriate prescription of antibiotics before blood was drawn for culture, and intermittent and low-load bacteremia. Although dental disease and treatment have been reported to be associated with IE [22], none of the patients with periodontal disease-associated SPE was diagnosed with IE. The reason for the lack of correlation between periodontal disease-associated SPE and IE is not clear. However, one reason is that the patients with periodontal disease-associated SPE did not have risk factors for IE, including presence of a prosthetic heart valve, prior IE, and complex congenital heart diseases, except for one patient [23].

In contrast to all cases of SPE in general, periodontal disease-associated SPE was successfully treated with antibiotics in addition to periodontal surgery. No patient died due to periodontal disease-associated SPE, suggesting that periodontal disease-associated SPE has a good prognosis.

In our case, *P. micra* was isolated as the causative organism of SPE, as confirmed by the positive blood culture result. *P. micra* is a Gram-positive anaerobic coccus normally found in the oral cavity, respiratory system, gastrointestinal tract, and female genitourinary tract. Originally known as *Peptostreptococcus micros*, the Gram-positive anaerobic coccus was reclassified as *P. micra* in 2006 [24]. *P. micra* is one of the bacterial species most frequently isolated from the infected root canals of teeth with chronic apical periodontitis [25]. *P. micra* has also been implicated in meningitis [26], cervical and brain abscess [27, 28], IE [29], and spondylodiscitis [30]. In a literature review of 30 cases of *P. micra* infection, positive blood culture results were obtained in 11 cases (36.7%), including valvular infection (4 cases), vertebral infection (5 cases), infection of the meninges (1 case), and pulmonary and head and neck infection (1 case) [31]. In that study, 16 of 30 cases (53.3%) of *P. micra* infection showed the presence of risk factors for underlying diseases such as tooth extraction and periodontitis. To our knowledge, SPE due to *P. micra* has been previously described in one case, in which periodontal disease was not diagnosed by a dentist [32]. *P. micra* is usually susceptible to antibiotics such as penicillin, clindamycin, metronidazole, and imipenem; however, strains resistant to penicillin, clindamycin, and metronidazole have been reported [33, 34].

In conclusion, we report a rare case of periodontal disease-associated SPE in which *P. micra* could be the causative pathogen. It is likely that clinicians get confused when they encounter cases of SPE without major risk factors such as intravenous drug use, IE of the tricuspid valve, septic thrombophlebitis, and indwelling intravascular catheters. In such cases, periodontal disease could be an important source of SPE, and careful oral examination aimed at identifying the primary source of infection and causative pathogen, including anaerobes, may lead to appropriate and effective treatments for this condition.

## Abbreviations

CT: Computed tomography
IE: Infectious endocarditis
MRSA: Methicillin-resistant *Staphylococcus aureus*
MSSA: Methicillin-sensitive *Staphylococcus aureus*
*P. micra*: *Parvimonas micra*
SPE: Septic pulmonary embolism
